# A Researcher’s Self-Reflection of the Facilitation and Evaluation of an Action Research Project Within the Swedish Social and Care Context

**DOI:** 10.5539/gjhs.v7n3p105

**Published:** 2014-11-17

**Authors:** Zada Pajalic

**Affiliations:** 1 Faculty of Health Sciences, Department of Health, Nutrition and Management, Oslo, Norway; 2 Kristianstad University, Sweden

**Keywords:** reflection, action researcher, field

## Abstract

The AR’s (Lewin, 1946) assumption was that human beings exist in life spaces through interaction, and communication, and that knowledge development is the result of changes in cognitive structures through communication and involvement in research processes. And further, from the construction of new knowledge gained by learning from each other, comes the presumption to change. The participation is described as “citizen power” (Arnstein, 1969), which means being included in the development process. The degree of citizen power is based on partnership, delegated power and citizen control. All these steps are positioned at the highest level when ranking participation. Partnership means engaging and negotiating with the high level decision makers. The result of delegated power is apparent when the negotiations result in achieving leading decisions that influence a particular plan or programme.

## 1. Action Research (AR) as a Vehicle for Bringing About Changes in Welfare Social and Care Context

In AR, focus can be reflection concentrated on the first, second and third persons (Reason & Bradbury, 2008). In the case of the first person, focus is directed on investigating the direction of the person’s daily actions “from the inside” based on their experience and personal competence (Koch & Kralik, 2006; Reason & Bradbury, 2008). A person’s personal skills have a logical connection to their ability to act. Second person focus is on the dialogue situations when people meet each other in connection with common problems related to improving personal, and or professional practice (Bondas, 2010; Webb, 1989). The meeting assumes interaction through dialogue. Third person AR focuses on building bridges between large groups of people who cannot meet due to the long distances between them. An example of building bridges is a conference that creates dialogue featuring a mutual approach between people of different positions (Reason & Bradbury, 2008).

First-person AR is described as the exploration of what the researcher brings to the research process and his/her interaction with the field. Burges (2006) described that being a facilitator was like moving between, confusion clarity, and anxiety due to not having control over the research process (Burgess, 2006). Self-reflection is described as the catalyst for learning oneself, being personally exposed and vulnerable (Marshall & Mead, 2005). It is important for the learning and the research process that the researcher personally investigates their own feelings by describing critical incidents in research (Heen, 2005). Taking the attitude of inquirer refers to the quality process that enables researchers to be aware of and articulate the complex process of interpretation, reflection and action. The process involves curiosity, willingness to articulate and explore multiple ways of accumulating knowledge during the research process while a self-reflective process enhances quality and validity (Marshall & Reason, 2007). The challenge is how to gain feedback and evaluate the research process and how participant responses might be framed by the relationship developed during the research process (Marshall, 2004). The self-reflection process is described as an art and some persons have more skill for self-reflection than others. Like the mastery of any skill it takes time, and the practice of self-reflection as a skill requires learning to be natural and open thereby bringing the research process to its full complexity and richness (Chiu, 2006; Taylor, 2004).

The outcome of any AR process depends on what happens at the beginning, for example the way of access to the adopted research field and its establishment, communication and at how high a level the participants are engaged. Establishing a contract is important for providing a clear sense of purpose at an early stage with focus on negotiation around a research process proceedings, relationships, roles and responsibilities. Facilitation creates conditions in which participants can contribute and thereby feel creative (Wicks & Reason, 2009). In order to implement change through AR, it is important to consider not only the bottom-up approach representing practitioners and service users, but also from the top-down perspective i.e. at management level. Both perspectives are required to ensure the balance of power needed to achieve progress. Relationships between management representatives and practitioners can be sensitive and stressful which requires that they are built up over time (Stringer & Genat, 2004). The purpose of involving people who are at management level (top-down) can be that persons at this level can facilitate future developments in practice. The interplay between these two perspectives is essential if change is to be possible or impossible to implement (Bondas, 2010). Involvement of a bottom-up perspective can be described as empowering strategies such as: information shared between participants is shared in order to achieve a better understanding of the problem area to be developed and to form good internal relationships between the participants (Kitson, 2009). Also external relationships should be established, such as with local and regional stakeholders and decision makers, to help increase awareness and generate insights by “social learning” in order to find new ways to solve specific problems (Meyer, 2004; Pajalic, Persson, Skovdahl, & Westergren, 2012; Pajalic & Westergren, 2013). Participants are encouraged to systematically investigate areas of their practice that are in need of development, and to plan for how to develop and transform these areas. The level of participation is contextualized as “participant power” meaning delegated power by negotiating the optimal conditions for positive development (Arnstein, 1969).

## 2. Purposes Behind the Start-Up and Facilitation of an AR Project

This research project was one AR project in a region of the province of Scania in the southern part of Sweden. All AR projects originate as the result of a decision at the highest political level (Månsson, 2007). The decision leading up to this AR project plan was to bring about changes and strengthen collaboration between different social and care organisations with the aim to provide more consumer social care and care services. The care consumers should be in the centre (Månsson, 2007; Winberg Ingvar, Lundgren Hanne, Liselotte, & Elisabeth, 2007). Further it was advocated from the highest political level that care should offer client participatory service. The client participatory service should be achieved through a constructive dialogue between care users with health care professionals as a catalyst towards decisions related to common practice changes (Winberg Ingvar et al., 2007). These changes mandate more direct forms of care consumer participation. For these care consumers participation in their own care offers the possibility of significant effects on both the care quality and the safety aspects. The fact that any organisational changes should be connected to research resulted in the creation of a cross-professional Research Platform for Development of Närsjukvård (Petersson & Blomqvist, 2011) with a professor as main facilitator, and an expert in AR as a research coordinator (Winberg Ingvar et al., 2007). With the AR approach, all research projects shall be based on ideas taken from practice in the targeted geographical area. A common assumption in the planning stage was to use the AR care givers and care consumers experience and knowledge which was considered important for achieving progressive practices in different care organisations.

## 3. How the AR Project Was Started?

This AR study was set up to collaborate with an organisation in one of the sex municipalities with the responsibility for food distribution (FD) to older people living in the municipality in their own homes. When I entered the research field I had no pre-formulated research questions (Pajalic, Springett, & Dychawy-Rosner, 2007). Noteworthy was that an organisation in one municipality had initiated the present research and it was natural from the beginning that the study would concern elderly persons in the municipality living in their own homes and their food intake. For this reason I performed an extended literature review with the aim of investigating what was published, nationally and internationally in this field. The literature review was made using field work and took about six months. From the beginning, two strategies determined the starting point. Both strategies contributed to the starting position of for the research with focus on learning to understand FD as an organisation, all those involved in the organisation, and its influence on its receivers i.e. the elderly persons receiving food supplies. The inclusion of all the perspectives of those involved in the organisation was important as they were interacting and dependent on each other. The meaning was that these perspectives should be a vehicle for future changes in the municipal FD organisation (Pajalic, 2013b).

Keeping in mind the aim research approach, I initiated dialogue with presumptive participants, ie the involved personnel about the ways they could be engaged in and contribute towards the research. Inclusion of these personnel as participants in the study from the beginning of the research process was important in order to define the research questions. The negotiation resulted in a common decision that I should be given the primary responsibility for data collection and analysis. During the field work, and after dialogue with professionals, it was decided that the qualitative methodological approach would be suitable as a starting-point. The intention with using AR was that participants would have the opportunity to reflect on development areas in order to come up with proposals for new solutions to use in practice. It was important that these proposals came from the participants and not from me as the researcher. An important requirement was that the participants felt motivated to participate in the study and willing to share their expertise related to municipal FD. Furthermore, active involvement by the participants was required in order to validate the preliminary results of the data analyses which were performed with the aim of avoiding naive conclusions and non- relevant recommendations. The participants’ involvement in the project was evaluated continuously, for example after each observation occasion and interview, and at the reconnection of the preliminary results and after completion of the research project (Pajalic, 2013a, 2013c).

## 4. What My Background Could Add to an AR Process?

From an AR first -person perspective, I reflected during the research process over what in my own background may be of interest in relation to my interaction with the study participants. My experience of coming into a new context by living in Sweden and starting a new life there, with all the adversities and progress this involves, has changed my view when it comes to observing situations and interactions between the participants in this study. My belief was that I had become more humble and sensitive to signals of sadness than I was before I came to Sweden. My personal development began through re-education, learning a new language, and culture and further getting to grips with new written and unwritten norms and working methods in the Swedish public sector. My overall reflection was that there was no lack of material support for citizens in various ways but each person had a personal responsibility to access any form public support. The message here was that each individual needs to be relatively self-sufficient and prepared to take personal initiative towards resolving their own problems. Further, they should always try to gather necessary information by them self and work hard at this. In Sweden, and the other Scandinavian countries, there is an unwritten rule that can be summarised as indicating that no one should believe that they are better than another person. My reflection is that this kind of low self-image influences the whole public practice and Swedish behaviour, including my own after 22 years of living in this cultural context. During this time I had obtained Swedish registration as a nurse and as a midwife, I have achieved both a bachelor and master’s degree in nursing and PhD in medical sciences with focus on health and nursing. I have been employed since 1995 with three public sectors as employers: municipality, county council and latest by the state. I have worked as a registered nurse/midwife at various units including hospitals, nursing homes and patient’s own homes in two municipalities. I have also worked as a staff manger. For six years I was politically engaged as a juror on a municipal political committee and as a juror at county council level, which means that I have the practical experience of being a decision-maker in political questions at the highest level of decision making at county council level. I drew the conclusion that my personal experiences could become an essential asset for the field work, for interaction with study participants and when gathering data. Furthermore, my personal experiences may have been useful as the guideline with which I facilitated the AR project from beginning to end.

## 5. What Was Important to Take Into Considerations When Approaching the Field?

The best way to approach this field was firstly to meet representatives from all levels who could affect my relationship with the participants. I could reason that I may have already been considered as part of this level and thereby might possibly be treated with suspicion, silence or indifference. Knowing that the first contacts with the participants involved in the research is crucial, I followed some general principles. Firstly, I introduced myself and my research as honestly as possible and emphasised that participants were welcome to ask questions. It was obvious to me that I should disclose my intentions with the research project especially as, participant observations can be contradictory. I thought that this was best because a researcher may withhold their personal reactions and intentions. I have tried to give brief, concrete explanations regarding what I was going to focus on during the participant observation. At the same time, I emphasised that it was they who were the experts and that they had valuable knowledge to contribute to the research project. In order to build social relationships and to be accepted by the participants, I presented the research purposes verbally to them on different occasions. I also used the strategy of treating participants with respect and showing interest in their work. In order not to create unnecessary disturbances and irritations to the participants in the field, I tried to follow the rhythms of their different activities during working hours. I was especially careful to be at the workplace at the same time as the staff began (07.30). I had noted early that there existed a collective norm that regulated staff punctuality. Late arrival was met with severe criticism or silence.

## 6. How My Research Role Changed During the AR Process?

My role alternated between facilitator, initiator, consultant and as both an in and outsider. Within the project I as researcher, was both an insider and outsider in the field and through the data collection. As an outsider I took the researcher’s role as an external agent, and as an insider I tried to become a part of the research field and become a catalyst and stimulator. My initial ambition was to adjust myself to the prevailing conditions. With AR in mind, I could recognise that I was dependent on the attitudes of the various personnel groups. My reflection was that conducting research from an AR approach was not isolated to one person but required reflections together with my supervisors and co-researchers at seminars with my AR colleagues and at AR conferences. During the whole project I continuously tried to focus on the participants’ confidence by always considering them as equals, which demonstrated an important task in an AR approach, namely providing democratic incitement (Pajalic et al., 2012).

## 7. Interpretation of the AR Process

My interpretation of why the current AR project was valuable and successful could be explained by the fact that the project was characterised by a high level of participant engagement, continuously expressed good relationships between all participants which appeared to indicate that the participants had trust in each other. This meant that trust, commitment and co-operation between me as facilitator and the study participants was seen to be established. In order to achieve a balance between the contact time length between myself and the study context, I sought to find a balance through being as sensitive as possible, trying always to “read between the lines” during each interaction situation with the intention of minimizing the risk of being “one of the subjects of the field” and to lose my objectivity. It was important that all the study participants should find their roles as experts in their area of practice and as co-researchers and that I was seen as an outsider with knowledge concerning systematic research methods that could help to give a perspective to problems and the possible solutions. I made an effort to see myself as the study participants saw me, which may have contributed to the positive progression of the research process. Capturing different perspectives was considered as a strength because the participants constituted a “critical mass of knowledge” which gave a broader picture of experiences related to municipal FD. My reflection was that the AR approach contributed to sustainable changes in the participants’ everyday knowledge related to the areas they wanted to change and their capacity to make change. Experiences gained from participation in the project gave new solutions in practice. The results returned to the participants were an evaluation and a basis for future improvements.

The most essential strength of the project was that it filled a knowledge gap that should offer understanding of the underlying experiences of municipal FD through the eyes of the participants. My perspective of understanding the context of FD through dialogue with participants was broadened and I became aware of the importance of self-reflection, since I had become a part in the creation of the research process. AR as an approach has brought strength to the study from many angles. I took the initiative by identifying possible research areas and discussing them with the participants. The consequence of this was that the final initiative for change came from them. This indicates that I was in a dependent position toward the participants’ commitment and consent. I also found that being a facilitator affected the AR process as I was engaged in research courses, seminars, conferences, reading the literature, and writing articles etc. Perhaps these interruptions influenced the interactive process with the participants and distanced me from the field, making it possible for me to not become too involved but left room for thought and reflection (Pajalic, Skovdahl, Westergren, & Persson, 2013).

The AR process can be looked at from three different levels, one that affected me as an AR researcher, the other affecting the study participants and the third that affected the research context. For me, clarity and credibility in the field was important for the creation of trust. My role as facilitator varied from taking more space and actively leading to taking a step back to allow the study to be more involved. There is no way to measure my own success in the field work but by evaluation of the fact that I was available in the field and how the research progressed may have given parameters to take in consideration. Anchoring the AR (in the minds of the study participants) is time-consuming because it is about promotion and facilitation of the processes created that were continually changing. The AR process promotes development based on the participants’ knowledge of practice linked to the areas that they wish to change. During the study, new solutions for practice offered by the participants were identified rather than solutions from me as action researcher. The participants were self-motivated to take part in the study. The study participants’ involvement in the AR study can be seen as an educational journey. After each observation and interview opportunity they reflected on what they had been through and how their involvement affected their way of looking at their daily practice (Pajalic, 2013d).

## 8. Conclusion

To achieve change often included involved contexts with a leadership that is open to change. Criteria for willingness to change were: that all participants are involved based on their individual circumstances; that they need to focus on their context and those that were part of the research process, action and evaluation. To achieve sustainable change requires knowledge and evidence in order to effect change in the “right way”. The conditions may, for example, be given by an organisation that needs to act as an enabler of change. Motivation and to feel motivated towards change depends if the change is perceived as meaningful. To realise change and sustainable development needs space for both action and encouragement. Engagement in meaningful collaborative work is essential for sustainable development where the participants represent a genuine critical mass of knowledge with first-hand experience. These experiences are important for promoting a feeling of confidence that realistic change is possible. The various professionals involved in municipal FD should have the possibility to collaborate on a more extensive level as their practical knowledge is needed for future organisational development.
